# Supplementary figures and images for: Lymphocyte deficiency alters the transcriptomes of oligodendrocytes, but not astrocytes or microglia
Source: PLoS One. 2023 Feb 24;18(2):e0279736. doi: 10.1371/journal.pone.0279736 (PMC9956607; doi:10.1371/journal.pone.0279736)

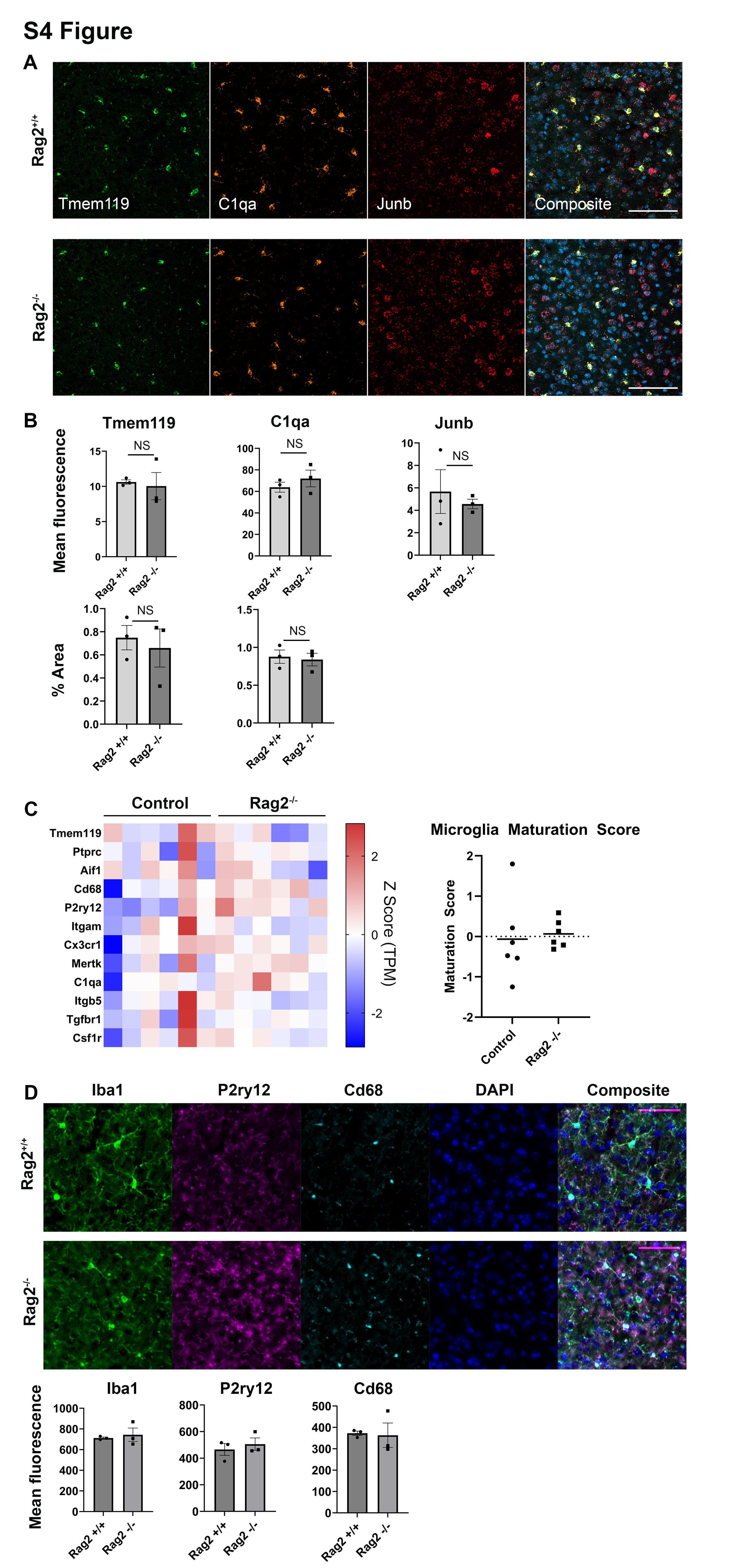

Supplement: S1 Fig — A) Example RNAscope images of microglial markers Tmem119 (green), C1qa (orange), and Junb (red) and composites including DAPI (blue) in the cerebral cortex. Top: Rag2+/+ immunocompetent; bottom: Rag2-/- immunodeficient. Scale bar = 100 μm. B) Quantification of RNAscope based on fluorescence intensity (top row) or area (bottom row) of microglial genes Tmem119 (Mean fluorescence: p = 0.79, mean[control, KO] = 10.6, 10.0, SD[control, KO] = 0.54, 3.34; Area: p = 0.68, mean[control, KO] = 74.9%, 66.0%, SD[control, KO] = 18.3, 28.6), C1qa (Mean fluorescence: p = 0.43, mean[control, KO] = 63.9, 72.0, SD[control, KO] = 8.0, 13.5; Area: p = 0.77, mean[control, KO] = 87.7%, 83.9%, SD[control, KO] = 15.2, 14.4), and Junb (Mean fluorescence: p = 0.63, mean[control, KO] = 5.67, 4.56, SD[control, KO] = 3.37, 0.74) from Rag2-/- and control mice (n = 3 KO, 3 WT). Error bars = SEM. C) Expression of microglia marker genes. Left: Heatmap of expression of 12 microglial marker genes, shown as transcripts per million (TPM) with z-score normalization across all samples, defined as (expression in the sample—the average expression across all samples)/standard deviation. Right: Microglial maturation score quantification, defined as the average z-score across all genes for each sample (p = 0.81, mean[control, KO] = -0.065, 0.065, SD[control, KO] = 1.03, 0.35). D) Immunostaining of microglial proteins. Top: representative images of Iba1, P2ry12, and Cd68 from Rag2+/+ and Rag2-/- mice. Scale bar = 50 μm. Bottom: Quantification of fluorescence intensity for Iba1 (p = 0.67, mean[control, KO] = 712, 743, SD[control, KO] = 15.8, 122.4), P2ry12 (p = 0.57, mean[control, KO] = 466, 506, SD[control, KO] = 77.1, 80.6), and Cd68 (p = 0.89, mean[control, KO] = 373, 363, SD[control, KO] = 17.6, 99.1). Error bars = SEM. (TIF) [file pone.0279736.s004.tif]

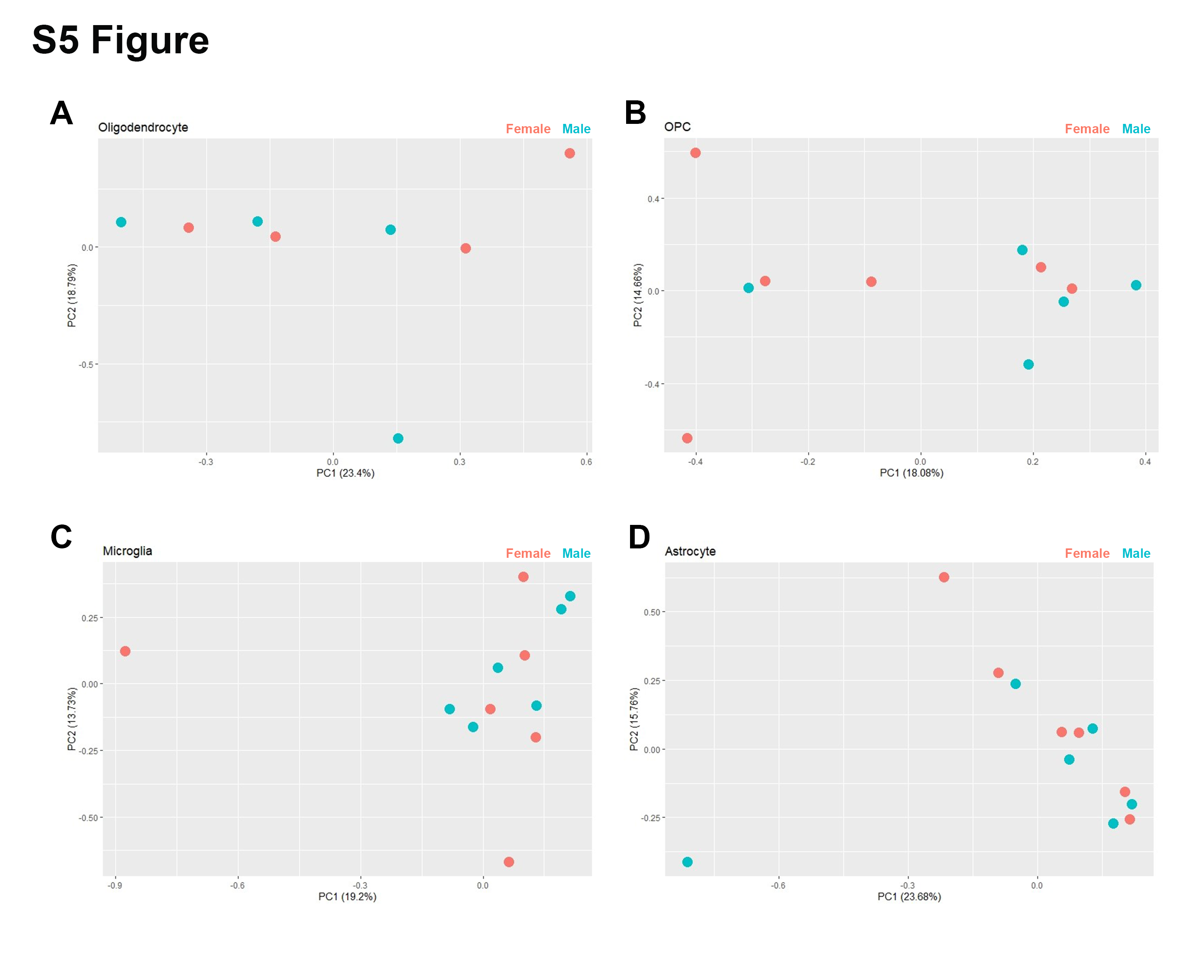

Supplement: S2 Fig — PCA of RNA sequencing data from glia from Rag2-/- and control mice. Red = female, blue = male. (TIF) [file pone.0279736.s005.tif]
