# Supplementary material for: Lymphocyte deficiency alters the transcriptomes of oligodendrocytes, but not astrocytes or microglia
Source: PLoS One. 2023 Feb 24;18(2):e0279736. doi: 10.1371/journal.pone.0279736 (PMC9956607; doi:10.1371/journal.pone.0279736)
Supplement: Attachment — Submitted filename: Reviews.docx [file pone.0279736.s006.docx]

**Synopsis:** The authors set out to investigate whether in the healthy brain, lymphocytes play a role in the transcriptomic phenotype of glial cells. The study has solid foundation in the idea that adaptive immune changes correlate with changes to glial cells in various disease states. To answer the question the authors use lymphocyte (Rag2^-/-^) deficient mice in naïve states as well as control mice and conduct RNA sequencing on acutely purified microglia, astrocytes, OPCs, and oligodendrocytes. The sequencing reveals that once lymphocytes are eliminated there are no transcriptomic differences in glia except for modest differences seen in the oligodendrocytes, though gross morphology of WM remains the same. Although there is space and need for a publication of this kind, as it stands the publication is lacking clarity as to the overall impact of the findings and the authors must expand on the topic (see below).

- Given there were no differences or very slight differences found (in the case of the OLs) all analysis possibilities should be exhausted.
  - Authors provide numbers of mice in each group but not specific numbers of male and female mice used. Was the transcriptomic data striated by sex? Did this elucidate any differences?
  - Further, the immunopanning for oligodendroglia is conducted using two antibodies for OPCs and one for Mature OLs. Although O4 classically marks “OPCs”, it more specifically marks the stage of the lineage from committed OPCs to pre-myelinating OLs. Have the Authors tried separating the O4 and PDGFRα panned cells? Did this make a difference transcriptomically?
- Figure 2A: Could the authors run a PCA on the data to get an overall view of gene expression changes between groups?
- Figure 2B: where were the images taken (more specifically)? Bregma? Were other myelin-rich regions checked? Can the authors include a quantification?
- Lines 248-259: Microglial “maturation” occurs during developing stages when primitive macrophages progenitors migrate to the developing neural tube and become microglia. From that point on microglia clonally expand in response to insult or injury. The use of “maturation” when referring to microglia makes little sense, it must be clarified.
  - Figure 3A: if the authors had already shown no difference between RAG negative and positive mice in the RNA seq of microglia, there is little need for the RNA scope to be included as a main figure.
  - Lines 254-259: Microglia are antigen-presenting cells that express MHCII. The comparison between their Rag2 negative and a lymphocyte-deficient MHCII KO model (Pasciuto) seems weird as one would affect microglia and the other wouldn’t. Please explain rationale.
- Lines 223-245: Were these upregulations confirmed via RNA scope or even immunofluorescence? Are these upregulations physiologically significant given gross myelin patterns appear normal? Please refer to comment on Figure 2B: can the authors provide further staining of myelinated regions w/ quantifications?
- Lines 272-282: In the GSEA the authors are again comparing a model which involves knocking out a microglial protein (MHCII) to their model which leaves microglia intact. Please explain rationale.
